# Correction: Efficacy of Albendazole-Chitosan Microsphere-based Treatment for Alveolar Echinococcosis in Mice

**DOI:** 10.1371/journal.pntd.0004181

**Published:** 2015-10-29

**Authors:** The PLOS Neglected Tropical Diseases Staff

## Notice of Republication

This article was republished on September 29, 2015 to correct errors in the order of figures that were introduced during the production process. The publisher apologizes for the errors. Please download this article again to view the correct version. The originally published, uncorrected article and the republished, corrected article are provided here for reference.

## Supporting Information

S1 File Originally published, uncorrected article. (PDF)

S2 File Republished, corrected article. (PDF)
