# Supplementary material for: Correction: Efficacy of Albendazole-Chitosan Microsphere-based Treatment for Alveolar Echinococcosis in Mice
Source: PLoS Negl Trop Dis. 2015 Oct 29;9(10):e0004181. doi: 10.1371/journal.pntd.0004181 (PMC4626088; doi:10.1371/journal.pntd.0004181)
Supplement: S2 File — (PDF) [file pntd.0004181.s002.pdf]

PLOS | NEGLECTED TROPICAL DISEASES

RESEARCH ARTICLE

Efficacy of Albendazole-Chitosan Microsphere-based Treatment for Alveolar Echinococcosis in Mice

Maitiseyiti Abulaihaiti[1], Xiang-Wei Wu[1], Lei Qiao[1], Hai-Long Lv[1], Hong-Wei Zhang[1], Nasrul Aduwayi[1], Yan-Jie Wang[1], Xin-Chun Wang[2☯]*, Xin-Yu Peng[1☯]*

1 Department of Hepatobiliary Surgery, the First Affiliated Hospital, School of Medicine, Shihezi University, Shihezi, Xinjiang, China, **2** Department of Pharmacy, the First Affiliated Hospital, School of Medicine, Shihezi University, Shihezi, Xinjiang, China

☯ These authors contributed equally to this work.
* cwjwxc@163.com (XCW); pengxinyu2000@sina.com (XYP)

CrossMark
click for updates

OPEN ACCESS

Citation: Abulaihaiti M, Wu X-W, Qiao L, Lv H-L, Zhang H-W, Aduwayi N, et al. (2015) Efficacy of Albendazole-Chitosan Microsphere-based Treatment for Alveolar Echinococcosis in Mice. PLoS Negl Trop Dis 9(9): e0003950. doi:10.1371/journal.pntd.0003950

Editor: Timothy G. Geary, McGill University, CANADA

Received: February 8, 2015

Accepted: July 3, 2015

Published: September 9, 2015

Copyright: © 2015 Abulaihaiti et al. This is an open access article distributed under the terms of the Creative Commons Attribution License, which permits unrestricted use, distribution, and reproduction in any medium, provided the original author and source are credited.

Data Availability Statement: All relevant data are within the paper and its Supporting Information files.

Funding: This work was supported by the National Natural Science Foundation of China (NSFC nos. 81260412 and 81360453). The funders had no role in the study design, data collection and analysis, decision to publish and preparation of the manuscript process.

Competing Interests: The authors have declared that no competing interests exist.

Abstract

This study aimed to investigate the pharmacology and anti-parasitic efficacy of albendazole–chitosan microspheres (ABZ-CS-MPs) for established intraperitoneal infections of *Echinococcus multilocularis* metacestodes in an experimental murine model. Male outbred Kunming mice infected with *E. multilocularis* Metacestodes were administered with three ABZ formulations, namely, ABZ-CS-MPs, Liposome–Albendazole (L-ABZ), and albendazole tablet (ABZ-T). Each of the ABZ formulations was given orally at three different doses of 37.5, 75, and 150mg/kg, three times a week for 12 weeks postinfection. After administering the drugs, we monitored the pharmacological performance and anti-parasitic efficacy of ABZ-CS-MPs compared with L-ABZ, and ABZ-T treated mice. ABZ-CS-MPs reduced the weight of tissues containing *E. multilocularis* metacestodes most effectively compared with the ABZ-T group and untreated controls. Metacestode grown was Highly suppressed during treatment with ABZ-CS-MPs. Significantly higher plasma levels of ABZ metabolites were measured in mice treated with ABZ-CS-MPs or L-ABZ compared with ABZ-T. In particular, enhanced ABZ-sulfoxide concentration profiles were observed in the mice given 150mg/kg of ABZ-CS-MPs, but not in the mice treated with L-ABZ. Histological examination showed that damages caused disorganization of both the germinal and laminated layers of liver hyatid cysts, demolishing their characteristic structures after treatment with ABZ-CS-MPs or L-ABZ. Over time, ABZ-CS-MPs treatment induced a shift from Th2-dominant to Th1-dominant immune response. CS-MPs As a new carrier exhibited improved absorption and increased bioavailability of ABZ in the treatment of *E. multilocularis* infections in mice.

Author Summary

Alveolar echinococcosis (AE) is caused by the metacestode stage of Echinococcus multilocularis and is a rare but life-threatening disease. Albendazole is the most frequently used

anti-parasitic drugs in patients infected with AE. However, ABZ has only limited water solubility, and small differences in drug solubility may have a major influence on their absorption and resultant pharmacokinetic behavior. Chitosan microspheres as a new carrier increased ABZ absorption and bioavailability. The anti-parasitic efficacy of albendazole–chitosan microspheres (ABZ-CS-MPs) in mice infected with *Echinococcus multilocularis* was assessed. ABZ-loaded CS-MPs exhibited improved absorption and increased bioavailability in the treatment of *E. multilocularis* infections in mice compared with those treated with liposome–albendazole and albendazole tablet. ABZ-CS-MPs are in solid form that can be manufactured in capsules or tablets, which can be easily delivered to those in need, particularly those who are nomadic. Therefore, given the merits of low cost, portability, and simple manufacturing, ABZ-CS-MPs are a promising drug to treat alveolar echinococcosis.

Introduction

Alveolar echinococcosis (AE) is a serious helminthic zoonosis caused by the metacestode stage of *Echinococcus multilocularis*, this parasitic infection can lead to severe damage to the human liver, lungs and other organs, and is potentially lethal when left untreated [1]. AE is predominantly transmitted by foxes as the definitive host [2] and is highly endemic in areas of Xinjiang, Qinghai, and Ningxia Provinces, as well as in the northeast of Inner Mongolia in China [3, 4]. The major treatment option for AE is radical surgery accompanied by pre- and post-operative medical treatment. Operative resection of lesions is frequently incomplete because of the tumor-like proliferation of the parasite with diffuse infiltration of other structures [5]. In addition, most patients infected with AE are without specific symptoms at the early stage and often miss the opportunity for surgical therapy, usually resulting in a diagnosis of advanced AE disease [6]. In cases where surgery is impossible, medical treatment remains an effective option. Albendazole (ABZ) is the most common and effective antiparasitic drug for AE treatment. However, owing to its limited solubility in water and the resulting poor intestinal absorption by oral administration, ABZ has low oral bioavailability, plasma level, and liver distribution[7]. Moreover, ABZ usually involves the lifelong uptake of large doses of the drug, making the prevention of many severe adverse drug effects difficult [8].

The fact that both Th1 and Th2 cytokines and chemokines coexist in AE patients [9] suggests the presence of a mutual tolerance during the infection, which determines the growth of metacestode[10]. Many studies have demonstrated that Th1-dominant response is related to parasite clearance, which is characterized by the production of IL-2, TNF-α, and INF-γ, whereas Th2-dominant response is necessary for parasite resistance to protective immunity, which results in the release of IL-4, IL-5, IL-6, and IL-10. The susceptibility of the parasite to immune attacks is mainly determined by the balance between Th1 and Th2 cytokine profiles in the host [11].

Therefore, searching for novel candidate drugs for AE treatment is urgently needed to maximize antiparasitic effects [12]. In recent years, several investigators have attempted to test potential drugs with in vitro and in vivo experiments using ABZ alone or in combination with other antiparasitic agents, such as praziquantel, 2-methoxyestradiol, nitazoxanide, and mefloquine, which have different parasiticidal and parasitostatic effects on Alveolar Echinococcosis [13–16]. Nevertheless, the long-term efficacy of the above administrations is unconfirmed, and none of them are currently available for clinical use. Formulating ABZ with special carriers has

been a promising approach and may enhance its absorption and bioavailability if taken orally [17].

Chitosan (CS) is a biodegradable natural polymer with great potential for pharmaceutical applications owing to its biodegradability, biocompatibility, non-toxicity, and mucosal adhesion [18]. CS has been proven to increase the intestinal absorption and bioavailability of hydrophilic drugs substantially by increasing the paracellular permeability across the mucosal epithelia [19]. CS has also been shown to improve the dissolution rate for poorly soluble drugs, thus warranting further research on its bioavailability enhancement for drugs used in AE treatment. Our research group has previously completed the pharmacokinetic studies and preparation technology of albendazole-chitosan microspheres (ABZ-CS-MPs) and has opened a patent [20]. Furthermore, based on previous work, the antiparasitic efficacy of ABZ-CS-MPs against cystic echinococcosis in an experimental murine model was performed, and results showed that the efficacy of ABZ-CS-MPs was superior to liposome–albendazole(L-ABZ) and albendazole tablet (ABZ-T) [21]. Based on the above in vivo experiment, this study aims to determine the antiparasitic efficacy of ABZ-CS-MPs against secondary AE in a murine model.

Materials and Methods

Chemicals and drugs

All biochemical reagents were purchased from Sigma Co., unless otherwise stated, with reference standards of 97%-98%. Solvents (acetonitrile and methanol) used in the extraction and drug effect analysis were high-performance liquid chromatography (HPLC) grade and purchased from Sigma Co. L-ABZ was produced by the Department of Pharmacy, the Affiliated Hospital, Xinjiang Medical University (No. 130218). ABZ-T was a gift from Tianjin Schick Pharmaceutical Co., Ltd (TSKF, Tianjin, China, No. 12060336).

Preparation of ABZ-CS-MPs

ABZ-CS-MPs were prepared by emulsification cross-linking, with liquid paraffin as the oil phase, CS as the aqueous phase, Span 80 as the emulsifier, and glutaraldehyde as the cross-linking agent. The particle size distribution and average size of the MPs were analyzed using an optical microscope (Fig 1). The ratio of aqueous phase to oil phase was 1:5. The procedures performed were as follows (i) ABZ (100mg) was dissolved in acetic acid (1ml) in a flask and hand-shaken until completely dissolved. (ii)The flask containing the ABZ solution was placed in an ultrasonic bath, and distilled water (19 ml) was gradually poured in. CS was then rapidly added. (iii) The aqueous phase was stirred for 24 h using a magnetic stirrer at a low speed. (iv) For the oil phase, Span 80 was added dropwise to the liquid paraffin using an insulin syringe needle while rapidly stirring. (v) Then aqueous phase was then mixed with the oil phase in a flask under rapid stirring. (vi) Embedded glutaraldehyde in the above mixed solution was stirred for a while, and the supernatant of of mixed suspension was then centrifuged and removed. (vii) Petroleum was then added to the flask, which was centrifuged before supernatants were removed. $NaHSO_3$ was then added, and the flask was again centrifuged before supernatants were removed. (viii) Finally, absolute ethanol was added to the flask, and the solution was placed in a Petri dish, dried, and stored at room temperatures until use. The drug encapsulation efficiency was 80% and the drug loading efficiency was 11.4%.

Preparation of different ABZ doses

i. ABZ-CS-MPs were prepared by adding ABZ-CS-MPs powder to 0.9% saline solution shaking for 30min until completely mixed. Aliquots of aqueous suspension (0.2 ml) of

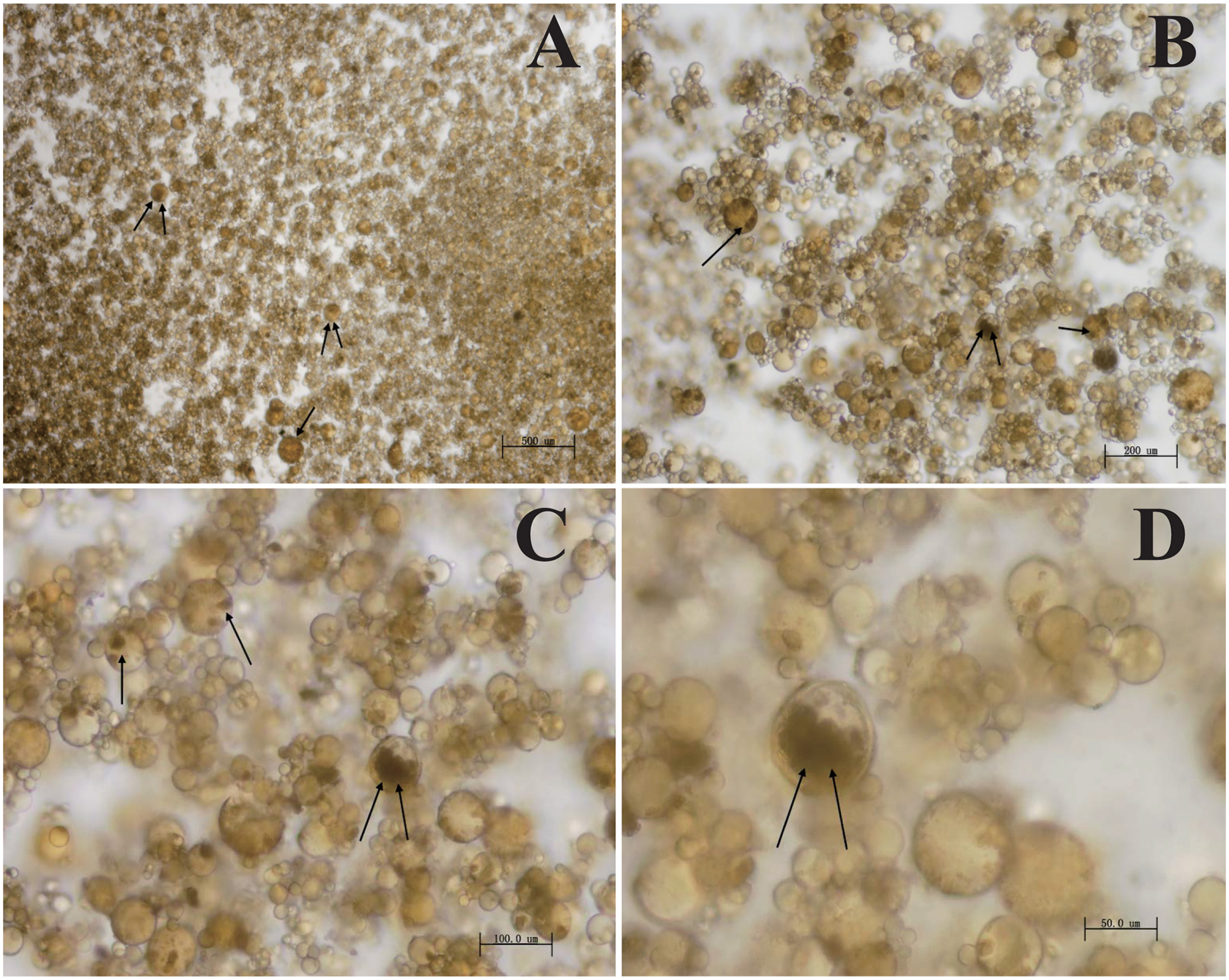

Fig 1. Albendazole chitosan microspheres (ABZ-CS-MPs). ABZ-CS was successfully entrapped by MPs (black arrows) (A: 40×, bar = 500 μm), (B: 100×, bar = 200 μm), (C: 200×, bar = 100 μm), (D: 400×, bar = 50 μm).

doi:10.1371/journal.pntd.0003950.g001

ABZ-CS-MPs were diluted to final concentrations of 37.5, 75 and 150 mg/mg. Each formulation was freshly prepared before administration.

ii. L-ABZ oral solution was mixed with 0.9% saline solution under shaking for 30min until completely mixed, Aliquots of aqueous suspension (0.2ml) of L-ABZ were diluted to final concentrations of 37.5, 75, and 150mg/mg. Each formulation was freshly prepared every day and stored at 4°C until use.

iii. ABZ-T was crushed and added to0.9% saline. Aliquots of aqueous suspension (0.2 ml) of ABZ-T were diluted to final concentrations of 37.5, 75, and 150 mg/kg. Each formulation was freshly prepared every day before administration.

iv. Control animals were orally administered with equal volumes of saline. ABZ (0.2 ml) was prepared for experimental and control groups and administered via intragastric inoculation.

Infection of *E. multilocularis* in mice

E. multilocularis metacestodes were originally isolated from naturally infected Mongolian gerbils for 24 weeks and used as source of *E. multilocularis*. Gerbils were euthanized by breaking their cervical vertebrae. Metacestode tissues were then removed from the peritoneal cavity and were minced well using scissors under sterile conditions. Minced tissues were suspended in sterile phosphate–buffered saline (PBS, pH 7.4) containing penicillin (20μg/ml) and then filtered through a mesh. The sediment containing fragments of vesicles, protoscoleces and calcareous corpuscles was washed with PBS three times to remove necrotic tissues and blood components. A 20% (sediment volume/total volume) suspension was prepared and stored at 4°C until use. The vitality of the protoscoleces was monitored using the methylene blue exclusion technique and the protoscoleces vitality obtained for this study was 90%. The number of intact metacestodes in 60μl was microscopically determined and 0.2ml of suspension containing 3,000 live protoscoleces was injected into multiple subcutaneous points at the lateral part of the abdomen on both sides of the mouse. Control mice received 0.2ml of saline in the same way. Three months (12 weeks) after infection, mice with successful inoculations were selected for further experimentation.

Experimental design and treatment efficacy

A total of 220 male outbred Kunming mice weighting 20±5 g were utilized for in vivo experiments, Animals were housed in a temperature-controlled (23 ± 1°C), light-cycled (12 h light/ dark cycle) room. Food and water were provided ad libitum. Animal experiments were carried out according to China Animal Welfare Guidelines (No. 2012–003–01).

At the 12th week after establishing experimental secondary AE in mice, the mice were grouped into five. Group 1 mice comprised 20 healthy mice as uninfected controls. The infected mice were randomly divided into four groups for oral administration of relevant treatments three times a week for 12 consecutive weeks. Group 2 mice comprised 20 infected mice as infection controls, only receiving 0.9% saline solution. Group 3 mice were administered 0.2 ml of ABZ-CS-MPs suspension. Group 4 mice received 0.2 ml of L-ABZ suspension. Finally, Group 5 mice received 0.2 ml of ABZ-T suspension. Mice in Groups 3, 4, and 5, were further categorized into 3 subgroups per group. Each subgroup was composed of 20 mice and was administered with 37.5, 75, or 150 mg/kg of ABZ. Each group of experiment data was analyzed according to their doses. Mice infected with *E. multilocularis* were euthanized 8 h following the final administration of ABZ suspensions. Blood samples from sacrificed animals were collected in heparinized plastic tubes immediately after euthanasia. Blood samples were centrifuged at 4000 × g for 15 min, and the recovered plasmas were stored at −80°C.

For assessing secondary infections, *E. multilocularis* vesicles were collected on the same days from sacrificed mice in Groups 2, 3, 4, and 5. Parasite samples on mouse tissues were carefully removed from the peritoneal cavity, and the parasite biomass was weighed for each mouse. The suppressive effect of cyst weight was calculated as the parasite inhibitory rate. The mean cyst wet weight was expressed as mean ± standard deviation (SD). The mean cyst weight of each ABZ-treated group was compared with that of the control group according to the following formula:

$$\text{AE vesicle inhibitory rate } (\%) = ((C - T)/C) \times 100\%$$

where C is the average wet weight of AE vesicle in the infected control group and T is the average wet weight of AE vesicles in the treatment group.

Morphological changes of *E*. *multilocularis* in mice

At the 24th week, all the mice from treated groups were euthanized and metacestodes that developed in the liver were photographed to assess the morphological alterations. The photographs were then compared among groups treated with ABZ-CS-MPs, L-ABZ, and ABZ-T, as well as the infected controls.

Histology and transmission electron microscopy (TEM)

A portion of the peripheral area of infected liver tissue with parasite vesicle samples was fixed in 10% neutral-buffered formalin at 4°C for 48 h. Pieces of infected tissues were dehydrated and embedded in paraffin wax, cut into several 2–5 μm-thick sections, and then stained with hematoxylin–eosin for histopathological examination.

A small piece of parasite material was processed for TEM. Briefly, metacestodes were fixed in 4% glutaraldehyde at room temperature for 2 h, followed by post-fixation in buffered 10% osmic acid for 1 h at room temperature. After dehydration, metacestodes were embedded in Epon 812 epoxy resin (Naguleswaran et al., 2006). Routine 0.5 μm ultrathin sections were stained with aqueous uranyl acetate followed by lead citrate before examination.

HPLC detection

1. Analytical methodology. 1.1 Analytical procedures. Pure standard compounds of ABZ-SO and mebendazole (MBZ) were purchased from Sigma Co. These were diluted with methanol to give 0.11, 0.22, 0.44, 0.80, 1.75, 3.50, 7.00, and 14.00 μg/ml standard solutions for standard curve calibration and to add to drug-free plasma samples to determine the recovery. Drug-free plasma samples (200 μl) were spiked with standards to reach the following final concentrations: 0.11, 0.22, 0.44, 0.80, 1.75, 3.50, 7.00, and 14.00 μg/ml. MBZ (1.0 μg/ml) was used as internal standard for the ABZ-SO analysis. After mixing for 15 s, plasma samples (200 μl) were added to 0.3 ml of PBS (pH 7.4) in a tube and spiked with 1 ml of MBZ solution. The tube was shaken on a slow rotary mixer for 10 min and centrifuged at 18,000 × g for 15 min. The supernatants were removed, and ethyl acetate (1 ml) was added to the previous tube, which was shaken and centrifuged following the above procedures. Sample tubes were then placed in an ultrasonic bath at 60°C until the supernatant was evaporated completely. Finally, 20 μL of this solution was injected into the chromatographic system.

1.2 Chromatographic conditions

Chromatography was performed on a Waters e2695 HPLC system (Waters Corporation, USA) with two pumps, an autosampler with a 50 μl loop, and a detector (2998 PDA) reading at 295 nm. A C18 reversed-phase column (5 μm, 250 mm × 4.6 mm, Waters, Sunfire, Part No: 186002560, Ireland) was used to analyze ABZ-SO. The calibration curves for each analyte, which were constructed by least-squares linear regression analysis, showed good linearity, with correlation coefficients of $\geq$ 0.995. The obtained ABZ-SO quantitation limit was 20 ng/ml, and inter-day precision were 5.33% (0.64 μg/ml), 5.11% (1.92 μg/ml), and 10.04% (5.76 μg/ml).

Mean absolute recovery percentages for concentrations ranging between 0.64 and 5.76 μg/ml (n = 5) were 103.04% (0.64 μg/ml), 99.82% (1.92 μg/ml), and 98.15% (5.76 μg/ml), with coefficients of variation (CV) of 5.4%, 3.8%, and 2.4%, respectively. The limit of quantification was defined as the lowest measured concentration with a CV $\leq$ 20% and accuracy of $\geq$ 70%. The flow rate was 0.78 ml/min. Under these conditions, the retention times were 4.433 and 9.681 min for ABZ-SO and MBZ, respectively.

Serum concentrations of IL-2 and IL-10

Capture enzyme-linked immunosorbent assay (Capture ELISA) was used to determine the concentration of cytokines IL-2 and IL-10 in serum of mice from all the experiment groups after necropsy was performed at weeks 18 and 22. IL-2 and IL-10 were used as marker cytokines for the Th1 and Th2 responses, respectively. Serum concentrations of IL-2 and IL-10 were determined by ELISA commercial kits (IL-2 and IL-10 high sensitivity ELISA kits, USCN, Wuhan, China), according to the manufacturer's instructions. The two cytokine-specific immunogens for IL-2 were Ala21 and Gln169, and those for IL-10 were Ser19 and Ser178 (USCN, Wuhan, China). The detection limit of the assay for both cytokines was 6.2 pg/ml.

Statistical analysis

Statistical analyses were performed using SPSS for Windows, version 20.0. Differences among the 10 experimental groups were analyzed using ANOVA, with P values of < 0.05 considered statistically significant. Parasite weights were compared using one-way ANOVA testing (two-tailed) and Dunnett's T3 test for multiple comparisons. Cytokine levels were compared by one-way ANOVA testing (two-tailed) and Fisher's least significant difference test. A non-parametric test (Kruskal–Wallis test) was used to compare pharmacodynamic data obtained from the three experimental groups.

Ethics statement

The animal study proposal was approved by the Institutional Animal Care and Use Committee of the First Affiliated Hospital, Shihezi University School of Medicine, with permit number 2012–003–01. All mouse experimental procedures were performed in accordance with the Regulations for the Administration of Affairs Concerning Experimental Animals approved by the State Council of the People's Republic of China.

Results

Reduced weight of parasite metacestode tissues after ABZ-CS-MP treatment

Table 1 shows the weights (mean ± SD) of parasite vesicles from untreated control mice and treated mice. The weights of parasite tissues from mice treated with ABZ-CS-MPs (0.176 ± 0.033, 0.123 ± 0.016, and 0.085 ± 0.029 g) were significantly less than those from infection control mice (1.565 ± 0.020 g, $P < 0.01$) or those from ABZ-T treated mice (0.496 ± 0.095, 0.433 ± 0.069, and 0.337 ± 0.063 g, respectively, $P < 0.01$). The parasite tissues removed from ABZ-CS-MPs treated mice weighed significantly less than those removed from ABZ-T treated mice in the 37.5 mg/kg subgroup ($F = 10.248$, $P = 0.036$), the 75 mg/kg subgroup ($F = 12.504$, $P = 0.012$), and the 150 mg/kg subgroup ($F = 8.142$, $P = 0.029$). ABZ-CS-MP therapy significantly reduced the growth of larval cysts, particularly in the 75 and 150 mg/kg subgroups. ABZ-CS-MPs had noticeable impact on the growth of the metacestodes in vivo ($P < 0.05$). For example, the inhibitory rates were 92.13% in the 75 mg/kg subgroup and 94.56% in the 150 mg/kg subgroup in terms of reduced weight of parasite tissues relative to the infection control group. However, the weights of parasite tissues showed no apparent differences between ABZ-CS-MPs treated groups and L-ABZ treated groups (0.156 ± 0.020, 0.147 ± 0.026, and 0.137 ± 0.026 g, $P > 0.05$), suggesting similar parasitostatic effects on the development of parasite tissue weights. Nevertheless, both drugs resulted in a significant reduction of parasite biomass compared to the ABZ-T treated group ($P < 0.01$).

Table 1. Comparative distribution of recovered parasite weight in *Echinococcus multilocularis*-infected mice treated with different doses of ABZ-CS-MPs, L-ABZ, and ABZ-T.

Group	Dose/Mg/kg-1	Number of survival mice	Weight of parasite vesicles	parasite inhibitory rate
ABZ-CS-MPs treated group	37.5	14	0.176±0.033	88.75%
	75	13	0.123±0.016	92.13%
	150	13	0.085±0.029	94.56%
L-ABZ treated group	37.5	13	0.156±0.020	90.03%
	75	12	0.147±0.026	90.58%
	150	13	0.137±0.026	91.23%
ABZ-T treated group	37.5	11	0.496±0.095	68.31%
	75	10	0.433±0.069	72.32%
	150	9	0.337±0.063	78.46%
Infection control group	0.2ml NS	13	1.565±0.020	0
Uninfected control group	0.2ml NS	16	0	0

doi:10.1371/journal.pntd.0003950.t001

ANOVA analyses (one-way, two-tailed Dunnett's T3 multiple comparison) showed significant differences among all ABZ formula-treated subgroups (for 37.5 mg/kg subgroup, F = 10.248, $P = 0.036$; for 75 mg/kg subgroup, F = 12.504, $P = 0.012$; and for 150 mg/kg subgroup, F = 8.142, $P = 0.029$).

Furthermore, our results showed negative correlations between the weights of parasite biomass from infected mice with incremental drug concentrations. As shown in Table 1, higher administered ABZ concentrations corresponded to significantly lower weights of parasite biomass in ABZ-CS-MPs treated groups ($P = 0.011$) and ABZ-T treated groups ($P = 0.067$). None of the mice exhibited aberrant behavior throughout the entire treatment regimens, suggesting that the treatments employed had no noticeable adverse effects.

Histological alterations after drug treatments

After oral administration of ABZ-CS-MPs and L-ABZ, the hydatid vesicles collapsed and the color of most lesions changed to yellow or cloudy white. The metastasis of hydatid vesicles was markedly suppressed. The sizes of liver surface vesicles significantly reduced in treated mice. Small calcified vesicles were observed on the liver surface (Fig 2A and 2B). As shown in Fig 2C, the liver surfaces exhibited large bubble-like vesicles, similar to tumor lesions. Fig 2D revealed that (1) alveolar hydatid expanded in the liver; (2) vesicles on the liver surface were in limpid hydatid fluid; and (3) the liver was filled with transparent or translucent *E. multilocularis* mass with small degenerated lesions.

Histology revealed that, in the ABZ-CS-MPs treated mice group, (1) the alveolar architecture of the parasite was severely collapsed; (2) the germinal layer had cellular swelling and reduced number of cells; (3) dissolved or partially disappeared laminated layer was observed; and (4) host inflammatory cells (such as histiocytes, lymphocytes, neutrophils, and eosinophils) invaded the crushed vesicles, severely collapsing the laminated layer (Fig 3A). In the L-ABZ treated mice group, we observed the following: (1) the laminated layer appeared to be broken and partially fragmented; (2) cells from the germinal layer exhibited partial defluvium, and laminated layer and coloboma appeared; and (3) inflammatory cells infiltrated the vesicles as well (Fig 3B). In ABZ-T treated mice group, the following phenomena were apparent: (1) partial shedding and dissolving of the cells of the germinal layer were observed to be identical; (2) the laminated layer was weakly stained pink; (3) it displayed as homogeneously dissolved in the laminated layer; (4) proliferation of fibrous tissue was visible as a red hyaline deformation,

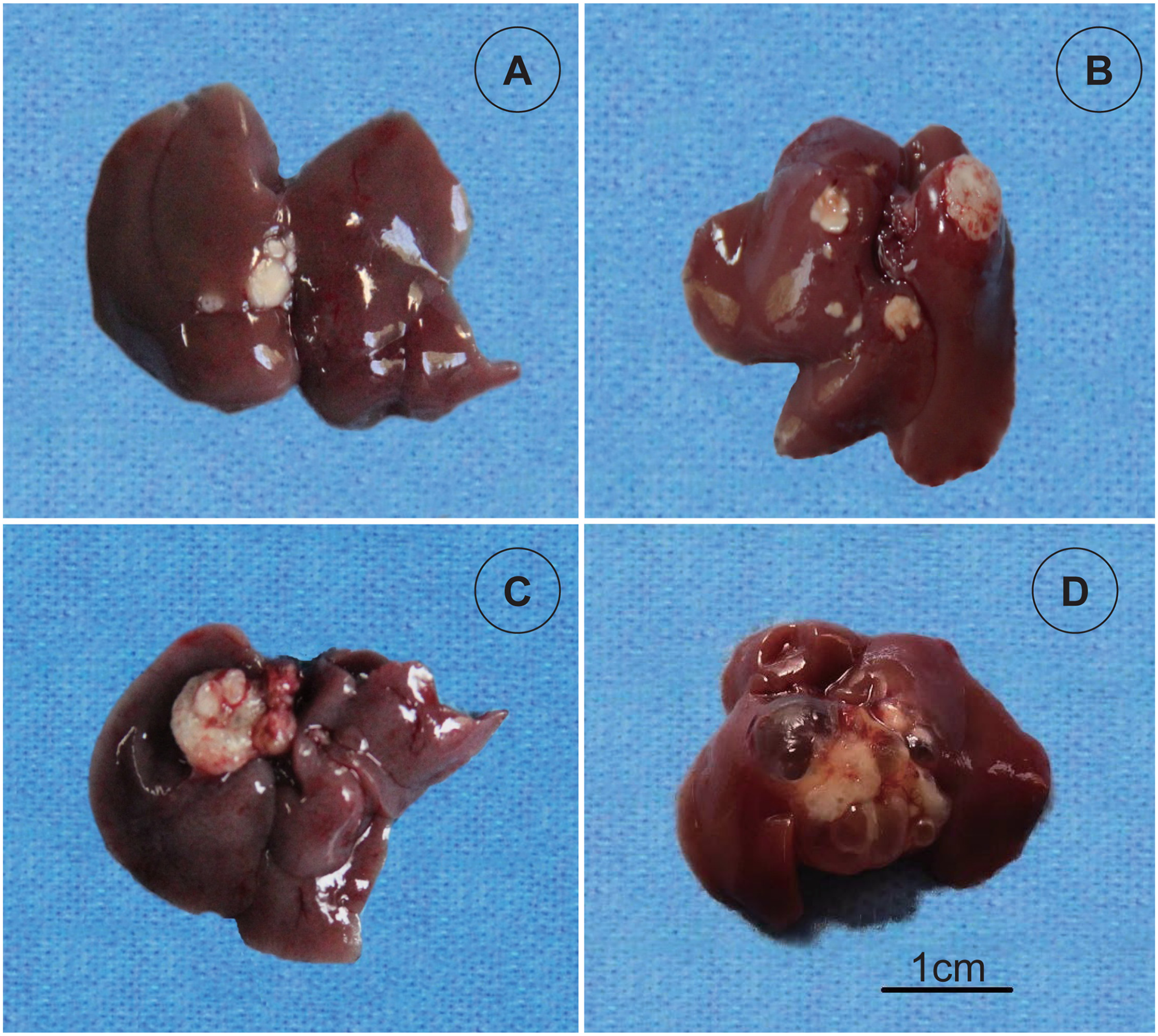

Fig 2. Hepatic lesions of mice infected with *Echinococcus multilocularis*. (A) ABZ-CS-MPs treated group: Note the highly suppressed vesicular development and yellow discoloration. Metacestodes were obviously calcified. (B) L-ABZ treated group: Note the degeneration of the metacestode biomass on the liver and cloudy white discoloration. (C) ABZ-T treated group: Note the large metacestode biomass. (D) Infection control group: Note the striped translucent vesicles and large metacestode mass almost occupying the entire liver. All infected livers shown were chosen from the 75 mg/kg subgroups.

doi:10.1371/journal.pntd.0003950.g002

and multifocal calcified tissues were observed; and (5) no inflammatory cells were observed (Fig 3C). In the infection controls, (1) a large number of cysts were observed, often surrounded by host connective tissues, which is a characteristic structure of *E. multilocularis*; (2) clear laminated and germinal layers were observed; (3) cells from the germinal layer were dense; and (4) laminated layer was uniform in thickness (Fig 3D).

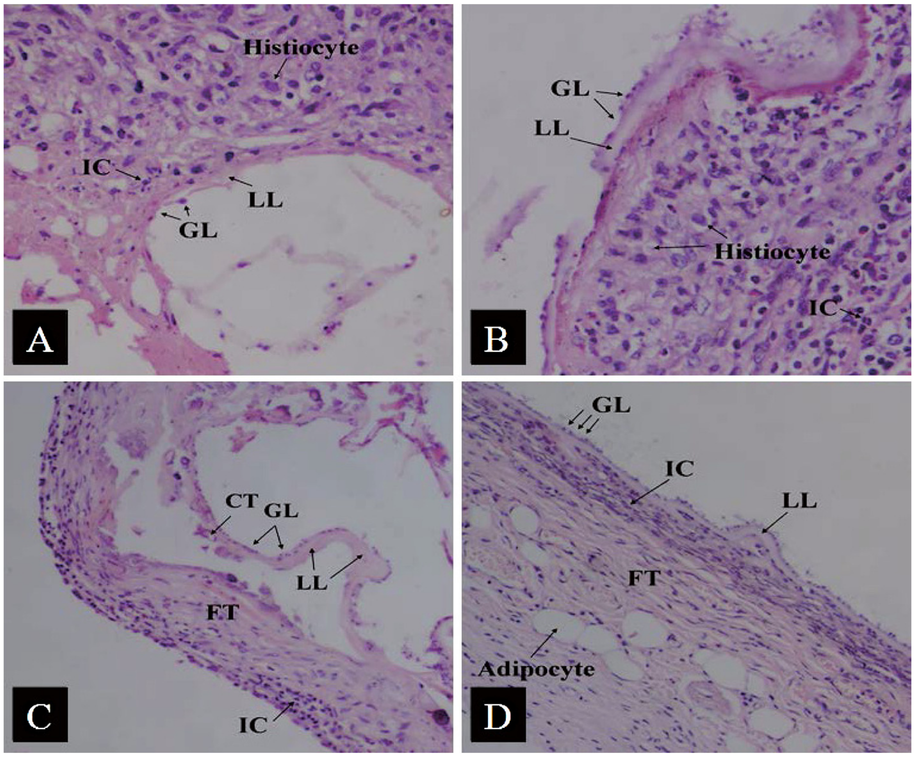

Fig 3. Histological sections of ABZ-treated and untreated metacestode tissue. (A) ABZ-CS-MP treatment: Note the high number of germinal layer cells dissolved and the partial dissolution of the laminated layer. (B) L-ABZ treatment: Note the thin, swollen structure of the laminated layer. (C) ABZ-T treatment: Note the irregularly thin laminated layer and the partially detached germinal layer cells. (D) Untreated infection control tissue: Note the intact, regular structure of the laminated and germinal layers. GL: germinal layer; LL: laminated layer; IC: inflammatory cell; FT: fibrous tissue; CT: calcified tissue; (200×).

doi:10.1371/journal.pntd.0003950.g003

The effects of different ABZ carriers on in vivo treatment were investigated using TEM, and we observed extensive tissue damage by parasites (Fig 4). Metacestodes from untreated controls presented complete structures with an intact and densely packed germinal layer, well-defined regular laminated layer, and clearly delineated microtriches, with the tegument adjacent to the laminated layer (Fig 4A). Distinct ultrastructural changes became evident and largely reduced microtriches, as well as pyknosis of mitochondria and cortical cells, were observed. The membrane structure of the germinal layer became obscure and loose (Fig 4B). Vacuole degeneration occurred in the germinal layer, and a sheet of laminated layer disappeared, making the layer uneven. Nevertheless, the cellular membrane structure of germinal layer was still clear and degeneration of somatic cells (including interstitial cells and fibroblasts) was apparent (Fig 4C). We observed fewer microtriches and a thin germinal layer with clear structure (Fig 4D).

High ABZ-SO levels in plasma in ABZ-CS-MPs treated mice

Mice infected with *E. multilocularis* were orally administered three different carrier types of ABZ at incremental doses of 37.5, 75, and 150 mg/kg. ABZ-SO, as a major metabolite in the plasma of treated mice, can be determined by HPLC. As shown in Table 2, experimental mice treated with ABZ-CS-MPs and L-ABZ exhibited significantly higher levels of ABZ-SO in the plasma compared with mice treated with ABZ-T. Non-parametric test (Kruskal–Wallis) revealed that plasma levels of ABZ-SO in ABZ-CS-MPs treated mice reached higher levels than

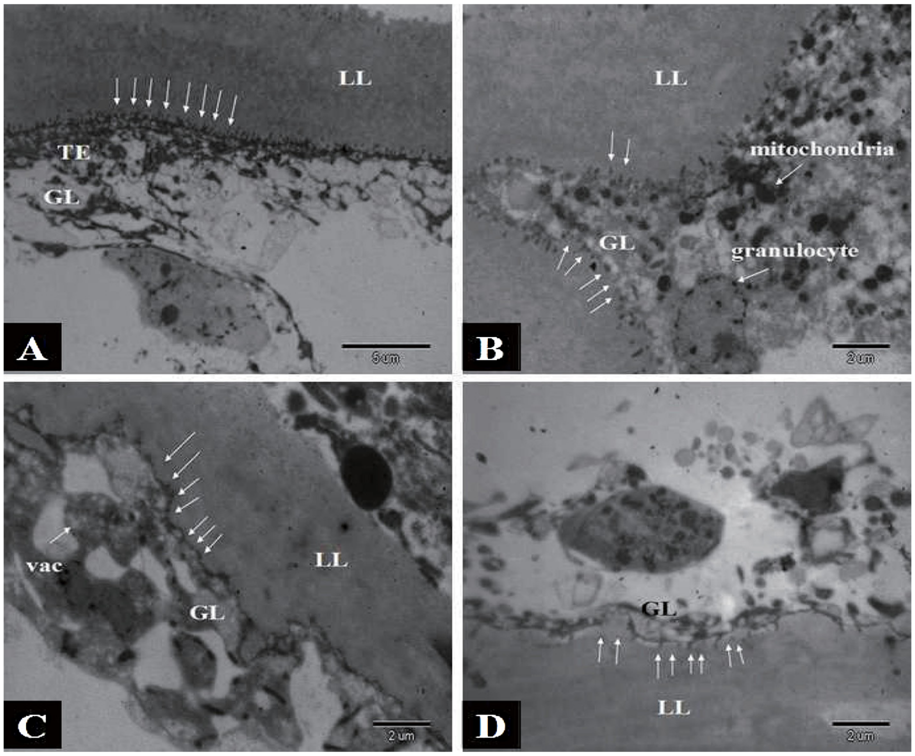

Fig 4. Transmission electron microscopy (TEM) analysis of drug efficacy in mice infected with *E. multilocularis* metacestodes and treated with ABZ-CS-MPs, L-ABZ, or ABZ-T. (A) Untreated infection control: Microtriches (white arrows) appeared intact and were densely arranged with clear structure of laminated layer (LL). A large number of microtriches protrude well into the laminated layer. Cortical cell (Cor) was present (black arrow). (B) and (C) ABZ-CS-MPs and L-ABZ treatment: Note the complete absence of microtriches at the tegumental-laminated layer interface (white arrows). Metacestode tissue appeared severely damaged, with regression of the germinal layer. Structural integrity of the germinal layer was destroyed. Laminated layer sheets were lacking, with vacuolization (vac) of the tissue in germinal layer. (D) ABZ-T treatment: Tegument of laminated layer lessened. A: (5000×, bar = 5 μm); B: (8000×, bar = 2 μm); C: (8000×, bar = 2 μm); D: (8000×, bar = 2 μm).

doi:10.1371/journal.pntd.0003950.g004

the ABZ-T treated mice in the 37.5, 75, and 150 mg/kg subgroups ($P < 0.001$). In the 150 mg/kg treated subgroups, the plasma levels of ABZ-SO in mice treated with ABZ-CS-MPs were higher than those in mice treated with L-ABZ ($P = 0.023$). No significant differences were observed in ABZ-SO plasma levels between ABZ-CS-MPs and L-ABZ treated mice in the 75 mg/kg subgroups ($P > 0.05$).

Serum levels of IL-2 and IL-10 cytokines

To relate the efficacy of different ABZ carrier forms and Th1/Th2 cytokine profiles, we measured serum levels of IL-2 and IL-10 cytokines as representative Th1 and Th2 markers, respectively. As shown in Table 3, serum levels of IL-2 in AE-infected control mice were significantly lower than those in uninfected control mice ($P < 0.05$). The levels of IL-2 in mice treated with ABZ-CS-MPs were elevated compared with those in infected control mice at week 18 postinfection ($P < 0.05$). At week 22 postinfection or 10 weeks after treatment, the levels of IL-2 in infected control mice were significantly lower than those in uninfected control mice ($P < 0.05$). Strikingly, mice treated with ABZ-CS-MPs showed increased levels of IL-2 compared with mice in the infected control group, as well as in the ABZ-T treated group ($P < 0.05$).

Table 2. Plasma concentration profiles of albendazole sulphoxide (ABZ-SO) in mice treated orally with ABZ-CS-MPs, L-ABZ, and ABZ-T, at doses of 37.5, 75, and 150 mg/kg.

Groups	Dose/mg.kg-1	Plasma concentration of ABZ-SO M(P25,P75)
ABZ-CS-MPs treated group	37.5	0.6785(0.5582,0.7823)
	75	1.1011(0.9218,1.2090)
	150	1.4035(1.1458,1.4910)
L-ABZ treated group	37.5	1.0410(0.8865,1.1957)
	75	0.8587(0.8305,1.4124)
	150	1.0275(0.8372,1.3401)
ABZ-T treated group	37.5	0.3141(0.2867,0.3317)
	75	0.4307(0.3827,0.4933)
	150	0.5409(0.4119,0.8135)
Uninfected control group	0.2ml NS	0
Infected control group	0.2ml NS	0

doi:10.1371/journal.pntd.0003950.t002

High levels of IL-10 were detected in the infection control group compared with the infection control group at week 18 postinfection ($P < 0.05$). By contrast, mice treated with ABZ-CS-MPs exhibited decreased levels of IL-10 compared with mice in the infected control and ABZ-T treated groups ($P < 0.05$). Furthermore, serum levels of IL-10 in mice in the infected control group remained higher than those in mice in the uninfected control group at week 22 postinfection. Mice treated with ABZ-CS-MPs showed decreased levels of IL-10 compared with mice in the infected control group and in the group administered with ABZ-T ($P <$ 0.05).

Discussion

ABZ has limited solubility in water, affecting its pharmacologic efficacy in clinical use. A slight increase in drug solubility has been shown to have major influence on intestinal absorption and resultant pharmacokinetic behavior [22]. Increasing the water solubility of ABZ using novel drug carriers is needed because orally administrated ABZ has not obtained a satisfactory effect for the medical treatment of AE patients. Nanoparticles have been studied as potential carriers of ABZ encapsulated in liposomes and have been reported to have promising results against AE [23,24]. In this study, we prepared CS MPs as a carrier of ABZ to produce ABZ-CS-MPs and tested their in vivo efficacy against AE in a mouse model.

E. multilocularis metacestodes in infected control mice appear to be large, with translucent vesicles filled with hydatid fluid (Fig 2D). ABZ-CS-MPs caused surface vesicles that developed on the liver to calcify and collapse significantly (Fig 2A). This finding suggests that this formulation has increased the hepatic availability of the drug. After treatment for 12 weeks, medical

Table 3. Serum concentration profiles (mean ± SD) for IL-2 and IL-10 cytokines in weeks 18 and 22 from *E. multilocularis*-infected mice treated with ABZ-CS-MPs or ABZ-T (75 mg/kg) and control, infection control groups.

Groups	IL-2		IL-10	
	Week18 pi	Week22 pi	Week18 pi	Week22 pi
ABZ-CS-MPs treated group	25.87±4.32	27.15±3.20	10.52±3.23	8.06±0.80
ABZ-T treated group	19.02±3.03	19.90±3.35	16.34±2.63	13.72±2.85
Uninfected control group	30.05±3.42	31.51±3.12	7.61±2.86	8.01±4.53
Infected control group	18.53±1.50	19.04±1.44	16.73±1.74	14.84±2.76

doi:10.1371/journal.pntd.0003950.t003

treatment using ABZ-CS-MPs is effective in terms of reducing parasite weights compared with untreated controls and the ABZ-T treatment group. By contrast, ABZ-T treatment resulted in only slightly reduced parasite weights. The enhanced suppressive effects toward the growth of *E. multilocularis* metacestode tissues may be closely related to the features of MPs that can achieve sustained drug release owing to improved intestinal absorption, which promotes prolonged exposure of local metacestode tissues to ABZ metabolites [25]. The cyst inhibitory rate in mice treated with ABZ-CS-MPs increased up to 92.13% in the 75 mg/kg dose subgroup and up to 94.56% in the 150 mg/kg dose subgroup, which were significantly higher than that in corresponding ABZ-T dose groups. This increased inhibitory rate is probably due to a synergistic effect of ABZ and the new carrier. The improved parasitostatic effect of ABZ is possibly caused by binding to parasite beta-tubulin, impairing cellular microtubular structures and inhibiting glucose uptake [26]. Incremental increase in the concentrations of ABZ-CS-MPs significantly reduced the wet weights of cysts in mice in a dose-dependent manner. This finding warrants further studies to observe differences in adverse effects among different doses of ABZ in experimental mice.

Histology examinations have shown that cellular structures are damaged in both the laminated and germinal layers of *E. multilocularis* metacestodes in treatment groups. After treatment with ABZ-CS-MPs or L-ABZ, parasite metacestodes were drastically damaged, showing dissolution in the germinal and laminated layers with structural discontinuity and breakage. Our results demonstrate that the cellular structure of germinal layer disappears in ABZ-CS-MPs treated mice but still exists in ABZ-T treated mice and L-ABZ treated mice, which may be due to the suitable permeability and high tensile strength of CS [27,28]. These properties of CS resulted in a better efficacy that may be attributable to its greater tissue penetration into cysts, causing further destruction of the germinal layer. Inflammatory cells in the pericystic layer infiltrate into the laminated layer, whose mechanism may involve the stimulation of immune responses [29]. The increased efficacy of ABZ-CS-MPs was also demonstrated by TEM. The ultrastructural changes observed through TEM include the loss of the characteristic multicellular appearance of the germinal membrane and the loss of most of the microtriches after ABZ-CS-MP treatment, whereas only minor damages are observed on the structure of parasite tissues in mice treated with ABZ-T. This finding confirms the anti-parasitic effect of the new formulation from a different point of view.

ABZ-CS-MPs achieved significantly higher plasma levels of ABZ-SO than ABZ-T in different dose subgroups, in which the 150 mg/kg subgroup had a plasma level of ABZ-SO higher than that of the corresponding subgroup in L-ABZ. The improved uptake and absorption of ABZ were obtained when this poorly water-soluble drug is incorporated into CS-MPs. In particular, CS-MPs may play a role in altering the mechanism of ABZ so that it easily adheres to the intestinal epithelium and passes the gastrointestinal barrier; this results in progressive degradation that may promote prolonged retention and action, increasing drug absorption and digestive tract release [30,31]. However, the specific mechanisms would need further physiologic investigations. A higher level of ABZ metabolites is also observed in mice treated with L-ABZ, possibly because of the usage of liposome entrapment of this drug. Nevertheless, our results presented are in agreement with those reported by Wen et al. [23].

Dynamic balance between anthelminitic and host immune response is likely to influence the evaluation of drug efficacy. IL-2 can enhance host immunity and inhibit parasitic growth, inducing a Th1-type immune response; by contrast, IL-10 inhibits cytokine production by Th1 cells while exhibiting increased serum levels with infection progression, resulting in mainly Th2-type immune response [32]. An immune imbalance indicated by a shift from Th1- to Th2-type host immune response occurs during *E. multilocularis* infection, which is beneficial in evading host immune attack, leading to disease development [33]. This study investigated

the effect of ABZ-CS-MP treatment on the balance between Th1- and Th2-driven cytokines by testing IL-2 and IL-10 levels. In the control group, serum IL-2 levels were significantly decreased in mice at week 18 and week 22 postinfection, whereas IL-10 levels were significantly increased compared with those of the controls ($P < 0.05$). Compared with the model control and ABZ-T treated groups, the production of IL-2, a Th1-type cytokine, significantly increased, whereas the level of IL-10, a Th2-type cytokine, decreased within the period of ABZ-CS-MP administration. Our data imply that the higher levels of IL-2 in the ABZ-CS-MPs treated group compared with the ABZ-T treated group may be responsible for more intensive inhibition of the cyst growth. ABZ-CS-MPs inhibited Th2 response, as evidenced by a suppressed total level of IL-10 cytokine after ABZ-CS-MP therapy, leading to a shift from Th2-dominant immune response to Th1-dominant immune response [34].

CS has been studied as a carrier for MP drug delivery. CS MPs are the most widely studied drug delivery system for the controlled release of drugs, antibiotics, anti-hypertensive agents, anticancer agents, proteins, peptide drugs, and vaccines [35,36]. One of the possible advantages of CS carrier over potential novel drugs for the treatment of AE is its non-toxicity. When CS formulations without ABZ (i.e., placebo) were orally administered to animals, a survival rate of 100% has been observed in experimental mice. In addition, no macroscopically differences were detectable between untreated and CS-MPs treated animals [37].

In conclusion, our results, including morphological and histological findings, pharmacological performance, and immunological parameters, demonstrate that ABZ-CS-MPs are a promising treatment for AE in mice. ABZ-CS-MPs improved bioavailability and absorption of ABZ and increased levels of ABZ-SO, which is a major metabolite of ABZ, in plasma. The chemotherapeutic effects of ABZ-CS-MPs and L-ABZ in our in vivo experiment were found to be similar. Our observations warrant pharmacokinetic studies and bioassays to assess the viability and non-viability of parasites conclusively and optimize ABZ-CS-MP treatment. Xinjiang Province, China has a high incidence of echinococcosis and shepherds are the most afflicted by this disease [38]. L-ABZ and ABZ oral emulsion are the main forms currently used clinically [39] and these forms are presently used as oral liquids, which have high storage requirements and short shelf life. Using these drug forms on shepherds who live a nomadic life in remote areas with poor access to health care is thus inconvenient. ABZ-CS-MPs are in a solid form that can be manufactured in capsules or tablets, which can be easily delivered to those in need, particularly those who are nomadic. Therefore, given the merits of low cost, portability, and simple manufacturing, ABZ-CS-MPs are a promising drug in the treatment of AE.

Acknowledgments

We acknowledge Professor Shi-Jie Zhang (The First Affiliated Hospital, Shihezi University School of Medicine) for providing the gerbils used in the present experimental work.

Author Contributions

Conceived and designed the experiments: XYP XWW XCW MA. Performed the experiments: XYP XWW XCW MA LQ HLL YJW. Analyzed the data: XYP XWW XCW MA HLL. Contributed reagents/materials/analysis tools: XYP XWW XCW NA YJW MA HWZ. Wrote the paper: XYP XWW XCW MA HLL.

References

1. Atanasov G, Benckert C, Thelen A, Tappe D, Frosch M, Teichmann D, et al. Alveolar echinococcosis-spreading disease challenging clinicians: A case report and literature review[J]. World J Gastroenterol 2013Jul; 14; 19(26):4257–61. doi: 10.3748/wjg.v19.i26.4257 PMID: 23864792

2. Lewis FI, Otero-Abad B, Hegglin D, Deplazes P, Torgerson PR. Dynamics of the force of infection: insights from Echinococcus multilocularis infection in foxes [J]. PLoS Negl Trop Dis. 2014 Mar 20; 8(3): e2731. doi: 10.1371/jounal.pntd.0002731 eCollection 2014. PMID: 24651596
3. Craig PS. Epidemiology of human alveolar echinococcosis in China[J]. Parasitol Int.2006; 55 Suppl: S221–5, Epub 2005 Dec 9. PMID: 16338167
4. Giraudoux P, Raoul F, Afonso E, Ziadinov I, Yang Y, Li L, et al. Transmission ecosystems of Echinococcus multilocularis in China and Central Asia[J]. Parasitology 2013 Nov; 140(13): 1655–66. doi: 10.1017/S0031182013000644 Epub 2013 Jun. PMID: 23734823
5. Reuter S, Jensen B, Buttenschoen K, Kratzar W, Kern P. et al. Benzimidazoles in the treatment of alveolar echinococcosis: a comparative study and review of the literature[J]. J Antimicrob Chemother, 2000Sep; 46(3): 451–6. PMID: 10980173
6. Nunnari G, Pinzone MR, Gruttadauria S, Celesia BM, Madeddu G, Malaguarnera G, et al. Hepatic echinococcosis: Clinical and therapeutic aspects. World J Gastroenterol 2012Apr 7; 18(13):1448–58. doi: 10.3748/wjg.v18.i13.1448
7. Daniel-Mwambete K, Torrado S, Cuesta-Bandera C, Ponce-Gordo F, Torrado JJ. The effect of solubilization on the oral bioavailability of three benzimidazole carbamate drugs [J]. Int J pharm. 2004 Mar 19; 272(1–2):29–36. PMID: 15019066
8. Brunetti E, Kern P, Vuitton DA. Expert consensus for the diagnosis and treatment of cystic and alveolar echinococcosis in humans[J]. Acta Trop, 2010 Apr; 114 (1): 1–16. doi: 10.1016/j.actatropica.2009.11.001 Epub 2009 Nov 30. PMID: 19931502
9. Hübner MP, Manfras BJ, Margos MC, Eiffler D, Hoffmann WH, Schulz-Key H, et al.Echinococcus multilocularis metacestodes modulate cellular cytokine and chemokine release by peripheral blood mononuclear cells in alveolar echinococcosis patients. Clin Exp Immunol 2006Aug; 145(2):243–51. PMID: 16879243
10. Vuitton DA. The ambiguous role of immunity in echinococcosis: protection of the host or of the parasite? Acta Trop 2003Feb; 85(2):119–32. PMID: 12606089
11. Vuitton DA, Gottstein B. Echinococcus multilocularis and its intermediate host: a model of parasite–host interplay. J Biomed Biotechnol 2010; 2010:923193. doi: 10.1155/2010/923193 Epub 2010 Mar 21. PMID: 20339517
12. Hemphill A, Müller J. Alveolar and cystic echinococcosis: towards novel Chemotherapeutical treatment options[J]. J Helminthol 2009 Jun; 83(2): 99–111. doi: 10.1017/S0022149X0928936X Epub 2009 Mar 19. PMID: 19296876
13. Dayan AD. Albendazole, mebendazole and praziquantal. Review of non-clinical toxicity and pharmacokinetics. Acta Trop.2003 May; 86(2–3):141–59 PMID: 12745134
14. Spicher M, Naguleswaran A,Ortega-Mora LM, Müller J, Gottstein B, Hemphill A. In vitro and in vivo effects of 2-methoxyestradiol, either alone or combined with albendazole,against Echinococcus metacestodes [J]. Exp Parasitol.2008 Aug; 119(4):475–82. doi: 10.1016/j.exppara.2008.02.012 Epub 2008 Mar 7. PMID: 18442817
15. Stettler M, Rossignol JF, Fink R, Walker M, Gottstein B, Merli M, *et all*. Secondary and primary murine alveolar echinococcosis: combined albendazole/nitazoxanide chemotherapy exhibits profound antiparasitic activity [J]. Int J Parasitol.2004 Apr; 34(5):615–24. PMID: 15064126
16. Küster T, Stadelmann B, Hermann C, Scholl S, Keiser J, Hemphill A. In Vitro and In Vivo Efficacies of Mefloquine-Based Treatment against Alveolar Echinococcosis [J]. Antimicrob Agents Chemother. 2011Feb; 55(2):713–21. doi: 10.1128/AAC.01392–10 Epub 2010 Dec 6. PMID: 21135182
17. Shuhua X, Jiqing Y, Mingjie W, Pieying J, Fanghua G, Junjie C, et al. Augmented bioavailability and cysticidal activity of albendazole reformulated in soybean emulsion in mice infected with Echinococcus granulosus or Echinococcus multilocularis [J]. Acta Trop. 2002 Apr; 82(1): 77–84. PMID: 11904106
18. Sinha VR, Singla AK, Wadhawan S, Kaushik R, Kumria R, Bansal K, et al. Chitosan microspheres as a potential carrier for drugs. Int J Pharm. 2004Apr 15; 274(1–2):1–33. PMID: 15072779
19. Thanou M, Verhoef JC, Junginger HE. Oral drug absorption enhancement by chitosan and its derivatives [J]. Adv Drug Deliv Rev. 2001 Nov 5; 52 (2): 117–26. PMID: 11718935
20. Xin Chun W, Xin Yu P. Preparation technology and chitosan microspheres component of Albendazole [P]. CN 102525947 A. 2012–07–04.
21. Liang W, Wang XC, Wu XW, Zhang SJ, Sun H, Ma X, et al. Efficacy of albendazole chitosan microspheres against Echinococcus granulosus infection in mice [J]. Zhongguo Ji Sheng Chong Xue Yu Ji Sheng Chong Bing Za Zhi. 2014 Jun; 32(3):188–92.(Article in Chinese) PMID: 25223052
22. Ceballos L, Elissondo C, Moreno L, Dopchiz M, Sánchez Bruni S, Denegri G, et al. Albendazole treatment in cystic echinococcosis: pharmacokinetics and clinical efficacy of two different aqueous

formulations [J]. Parasitol Res. 2008 Jul; 103(2):355–62. doi: 10.1007/s00436–008–0980-x Epub 2008 May 10. PMID: 18465143

23. Wen H, New RR, Muhmut M, Wang JH, Wang YH, Zhang JH, et all. Pharmacology and efficacy of liposome-entrapped albendazole in experimental secondary alveolar echinococcosis and effect of co-administration with cimetidine [J]. Parasitology. 1996 Aug; 113(pt 2):111–21. PMID: 8760312
24. Takeuchi H, Yamamoto H, Kawashima Y. Mucoadhesive nanoparticulate systems for peptide drug delivery. Adv Drug Deliv Rev. 2001 Mar 23; 47(1): 39–54. PMID: 11251244
25. Burjak M, Bogataj M, Velnar M, Grabnar I, Mrhar A. The study of drug release from microspheres adhered on pig vesical mucosa. Int J Pharm. 2001 Aug 14; 224(1–2):123–30. PMID: 11472821
26. Horton J. Albendazole: a review of anthelmintic efficacy and safety in humans. Parasitology.2000; 121 Suppl: S113–32. PMID: 11386684
27. Schipper NG, Olsson S, Hoogstraate JA, deBoer AG, Vårum KM, Artursson P. Chitosans as absorption enhancers for poorly absorbable drugs 2: mechanism of absorption enhancement [J]. Pharm Res.1997 Jul; 14(7):923–9. PMID: 9244151
28. Amiji MM. Permeability and blood compatibility properties of chitosan–poly(ethylene oxide) blend membranes for haemodialysis. Biomaterials. 1995 May; 16(8):593–9. PMID: 7548609
29. Godot V, Harraga S, Beurton I, Deschaseaux M, Sarciron E,Gottstein B, et al. Resistance/susceptibility to Echinococcus multilocularis infection and cytokine profile in humans. I. Comparison of patients with progressive and abortive lesions. Clin Exp Immunol. 2000 Sep; 121(3):484–90. PMID: 10971515
30. Thanou M, Verhoef JC, Junginger HE. Oral drug absorption enhancement by chitosan and its derivatives. Adv Drug Deliv Rev. 2001 Nov 5; 52(2):117–26. PMID: 11718935
31. Schipper NG,Vârum KM,Stenberg P, et al. Chitosans as absorption enhancers for poorly absorbable drugs.3: Influence of mucus on absorption enhancement. Eur J Pharm Sci. 1999 Aug; 8(4):335–43. PMID: 10425384
32. Zheng Y. Strategies of Echinococcus species responses to immune attacks: Implications for therapeutic tool development. Int Immunopharmacol. 2013 Nov; 17(3): 495–501. doi: 10.1016/j.intimp.2013.07.022 Epub 2013 Aug 20. PMID: 23973651
33. Vuitton DA, Zhang SL, Yang Y, Godot V, Beurton I, Mantion G, et all. Survival strategy of Echinococcus multilocularis in the human host. Parasitol Int. 2006; 55 Suppl:S51–5. Epub 2005 Dec 19. PMID: 16360335
34. Hübner MP, Manfras BJ, Margos MC, Eiffler D, Hoffmann WH, Schulz-Key H, et al. Echinococcus multilocularis metacestodes modulate cellular cytokine and chemokine release by peripheral blood mononuclear cells in alveolar echinococcosis patients. Clin Exp Immunol. 2006 Aug; 145(2):243–51. PMID: 16879243
35. Pilicheva BA, Kassarova MI, Zagorchev PI, Uzunova YI. Formulation and performance evaluation of betahistine dihydrochloride microspheres as sustained release system. Folia Med (Plovdiv). 2014 Jul-Sep; 56(3):187–93.
36. Dini E, Alexandridou S, Kiparissides C. Synthesis and characterization of cross-linked chitosan microspheres for drug delivery applications[J]. J Microencapsul. 2003 May-jun; 20(3):375–385. PMID: 12881117
37. Barrera MG, Leonardi D, Bolmaro RE, Echenique CG, Olivieri AC, Salomon CJ, et al. In vivo evaluation of albendazole microspheres for the treatment of Toxocara canislarva migrants. Eur J Pharm Biopharm. 2010 Aug; 75(3):451–4. doi: 10.1016/j.ejpb.2010.03.017 Epub 2010 Mar 27. PMID: 20347973
38. Zhou HX, Chai SX, Craig PS, Delattre P, Quéré JP, Raoul F, et al. Epidemiology of alveolar echinococcosis in Xinjiang Uygur autonomous region, China: a preliminary analysis[J]. Ann Trop Med Parasitol. 2000 Oct; 94(7):715–29. PMID: 11144813
39. Chai J, Menghebat Jiao W, Sun D, Liang B, Shi J, et al. Clinical efficacy of albendazole emulsion in treatment of 212 cases of liver cystic hydatidosis [J]. Chin Med J (Eng). 2002 Dec;11 l5(12):1809–13.
